# Assessment of lifestyle changes in combating the COVID-19 pandemic among people of Karachi, Pakistan

**DOI:** 10.2144/fsoa-2023-0227

**Published:** 2024-05-24

**Authors:** Mubashir Zafar, Tafazzul Hyder Zaidi, Nadira Hyder Zaidi, Muhammad Waqas Nisar Ahmed, Mahjabeen Shah, Umm e Habiba, Marrium Sultan Dar, Noor ul Ain, Fatima Shahid, Hiba Hamid Meer

**Affiliations:** 1 Department of Family and Community Medcine, College of Medcine, University of Hail, Hail, Kingdom of Saudi Arabia; 2 Community Medicine Department, Sindh Medical College, Jinnah Sindh Medical University, Pakistan; 3 Shaheed Zulfiqar Ali Bhutto Institute of Science & Technology, Karachi, Pakistan

**Keywords:** COVID-19 pandemic, Karachi, lifestyle changes, lifestyle habits

## Abstract

**Aim:** COVID-19 arose as a pandemic that wreaked havoc all over the world. Study determines lifestyle changes adopted by people of Karachi in response to COVID-19 pandemic. **Methods:** This was the cross-sectional study and carried out at public sector hospital and 218 participants were selected through random sampling method. **Results:** Lifestyle changes a statistically significant difference in nutrition (p = 0.000), physical activity (p = 0.000), workout (p = 0.000), smoking (p = 0.000), sleep hours (p = 0.000), and supplements (p = 0.000) before and during lockdown. Face masks were utilized by 38% of individuals, gloves by 19%, and social isolation was observed by 26%. 11% of participants did not follow any protocols. **Conclusion:** Study found significant differences in lifestyle habits such as diet, sleep, smoking and physical activity.

## Plain language summary

COVID-19 pandemic affect every day life of all people. This study results fond that there are different domain of quality life were affected. Nutrition, physical activity and smoking were associated with poor quality of life and these changes affect the mental and physical health of community people. There is need to social and medical support for people affected by COVID-19 pandemic.

## Abstract (plain language summary)

Total 44% of study participants were physically inactive.

Quality of life domain which common affected were physical inactive, poor sleep, fast food consumption and smoking.

Participants had increased likely to mental and physical distress.

On 12 December 2019, COVID-19 emerged in Wuhan city of China initiating a worldwide global pandemic [1]. The presence of COVID-19 can be confirmed by many symptoms ranging from mild or moderate illness which can even cause death [2]. Fever, coughing, weakness, pneumonia, headache, diarrhea, hemoptysis and dyspnea are the recorded symptoms [3]. In older people, symptoms could be more severe especially those having comorbidities can have more risk factors and fatality rates [4].

The COVID-19 has made dramatic changes in our daily life and mental health [5]. The implications of the pandemic of COVID-19 on both physical and mental health are severe. Home confinement and psychological distress may cause many harmful and unhealthy modes like inactive style, overeating, tobacco consumption and impaired sleep [6].

A globally conducted literature search showed that during the COVID-19 pandemic poor knowledge, attitudes, and practice skills are linked to a number of variables, including education level, occupation, income, gender, age, place of residence, work experience, religion, media use, marital status and race [7]. According to a meta-analysis, during the COVID-19 pandemic, the knowledge, attitude and practice prevalence in the Southeast and South Asia varied from 26.53% (Thailand) to 95.4% (Nepal), 59.3% (Turkey) to 92.5% (Pakistan), and 50.2 (Turkey) to 97% (Afghanistan), respectively [8].

Another systematic review conducted showed that individuals' body weight and overall food consumption during the COVID-19 pandemic increased by 51.0 and 57.2%, respectively [9]. According to a cross-sectional survey conducted during the COVID-19 pandemic, there were at least two detrimental alterations to the key lifestyle factors. The most prevalent personal unfavorable effects were an increase in snacking, sleep issues, a decrease in leisure-time physical activity, and active commuting to work. There were at least two significant improvements in the key lifestyle changes. Increased consumption of fruit, berries, and vegetables as well as a reduction in alcohol consumption were the most frequently observed positive improvements among individuals [10]. Another cross-sectional study during the outbreak showed GAD, depressive symptoms, and public sleep quality prevalence rates were 35.1, 20.1 and 18.2%, respectively [11].

The COVID-19-related nationwide lockdown has had a significant impact on the daily lives of Pakistani people, including significant changes to their sleeping patterns, eating habits, mental health and physical activity [12]. Karachi is the most populous city in Pakistan so there is a greater chance of exposure to the virus and, hence contracting the disease. To understand the impact of COVID-19 pandemic (2020) and associated determinants which impact the quality of life, functioning and overall HRQL of everyday life is therefore crucial due to the impact these outcomes may place on an already burdened health system, and because these said factors may contribute to an individual's uptake of health behaviors and risk of infection. Therefore, this study aims to evaluate lifestyle changes such as eating habits, physical activity and social distancing in fighting against COVID-19 among people of Karachi at Jinnah Postgraduate Medical Centre (JPMC) in Karachi, Pakistan. This research would contribute to the already existing literature and in providing information about those changes which a person can take in his behavior, habits, physical activity and diet to successfully combat COVID-19 in a heavily populated city.

## Methods

This cross-sectional study was carried out with a random sampling in the vicinity of public sector hospital in Karachi. The sample size was calculated using the openepi calculator. By keeping the confidence level as 95%, error limit as 5%, and anticipated frequency of positive changes in lifestyle as 14% [10] the sample size came out to be 185. By estimating a response rate of 84%, the sample size becomes 218.

Those participants were included in the study who were coming to outpatient department (OPD) of hospital and were willing to give consent while those were excluded who could not understand the questionnaire either in Urdu or English language. The participants were not provided with any monetary rewards or other tangible incentives. Data collection period from June 2022 to December 2022.

The Dependent variable for the study taken into account was the participant's lifestyle which included preferred diet, hours of sleep, physical activity, workout, smoking, preferred food groups and additional supplements while the independent variable was COVID-19 lockdown.

The data was collected by rotating a pre-tested self-administered questionnaire. To make it easier for the participants, the questionnaire was also translated into local language Urdu. It was distributed to the visitors coming to OPD. The questionnaire consisted of four sections. The first section consisted of informed consent and voluntary participation agreement. The second section requested demographic information including age, gender, education, and current status of his/her profession. The third section requested information on individual lifestyle before the pandemic of COVID-19 such as preferred diet, hours of sleep, physical activity, workout, smoking, preferred food groups and additional supplements. The number of hours of sleep for each patient was a factor in assessing the sleep quality. The fourth section requested information on lifestyle changes during the pandemic lockdown by asking similar questions which were asked in the third section.

Statistical data were analyzed by using SPSS software version 20.0 with a 95% confidence interval. The chi-square test was used to assess the difference in lifestyle before and during the COVID-19 pandemic lockdown. Statistics were considered significant if p < 0.05.

Ethical Approval was received from the Institutional Review Board of Jinnah Sindh Medical University before conducting the research. During this study, all the research misconducts were avoided and the rights and well-being of research participants remained protected.

## Results

Out of 218 participants, the majority i.e. 82.6% (n = 180) were 18–26 years old. There were 17% (n = 37) males and 83% (n = 181) females. Majority of the participants i.e., 63.8% (n = 139) were undergraduates and 72.9% (n = 159) were students (Table 1).

**Table 1.** Socio-demographic characteristics of our study participants.

Variable	n (%)
**Age (years)**	
18–26	180 (82.6%)
27–36	10 (4.6%)
37–46	13 (6%)
47–60	15 (6.9%)
**Gender**	
Male	37 (17%)
Female	181 (83%)
**Education**	
Primary	6 (2.8%)
Matriculation	9 (4.1%)
Intermediate	18 (8.3%)
Undergraduate	139 (63.8%)
Graduate	28 (12.8%)
Post-graduate	11 (5%)
Not formally educated	7 (3.2%)
**Current status**	
Student	159 (72.9%)
Homemaker	28 (12.8%)
Unemployed	9 (4.1%)
Self-employed	15 (6.9%)
Private sector employee	6 (2.8%)
Government sector employee	1 (0.5%)

A chi-square test was performed to compare the lifestyle before and during the COVID-19 lockdown. A Significant difference was found in the preferred diet (p = 0.000), hours of sleep (p = 0.000), physical activity (p = 0.000), workout (p = 0.000), smoking (p = 0.000), and additional supplements (p = 0.000) before and during the COVID-19 lockdown. However, there was no significant difference in the preferred food groups before and during the COVID-19 lockdown (p = 0.845) (Table 2).

**Table 2.** Self-reported lifestyle changes before and during the COVID-19 pandemic.

Variables	Before COVID-19 lockdown n (%)	During COVID-19 lockdown n (%)	p-value
**Preferred diet**			0.000
Healthy diet	56 (25.6%)	105 (48.1%)	
Fast food	10 (4.5%)	8 (3.6%)	
Both	152 (69.7%)	105 (48.1%)	
**Sleep (h)**			0.000
2–4	68 (31.1%)	6 (2.7%)	
4–6	28 (12.8%)	25 (11.4%)	
6–8	121 (55.5%)	79 (36.2%)	
8–10	53 (24.3%)	84 (38.5%)	
More than 10	8 (3.6%)	24 (11%)	
**Physical activity**			0.000
Mildly active	42 (19.2%)	96 (44%)	
Moderately active	117 (53.6%)	95 (43.5%)	
Highly active	59 (27%)	27 (12.3%)	
**Workout**			0.000
Yes	54 (24.7%)	36 (16.5%)	
No	111 (50.9%)	126 (57.7%)	
Sometimes	53 (24.3%)	56 (25.6%)	
**Smoking**			0.000
No	210 (96.3%)	206 (94.4%)	
Rarely	0 (0%)	3 (1.3%)	
Sometimes	5 (2.2%)	4 (1.8%)	
Regularly	3 (1.3%)	5 (2.2%)	
**Preferred food groups**			0.845
Dairy and meat	142 (65.1%)	172 (78.8%)	
Fruits	156 (71.5%)	171 (78.4%)	
Vegetables	174 (79.8%)	174 (79.8%)	
Grains and rice	178 (81.6%)	171 (78.4%)	
Beans and pulses	149 (68.3%)	141 (64.6%)	
**Additional supplements**			0.000
Warm water and drinks	49 (22.4%)	57 (26.1%)	
Vitamin supplements	45 (20.6%)	43 (19.7%)	
Calcium supplements	30 (13.7%)	34 (15.5%)	
Iron and zinc supplements	16 (7.33%)	19 (8.71%)	

**Figure 1.**
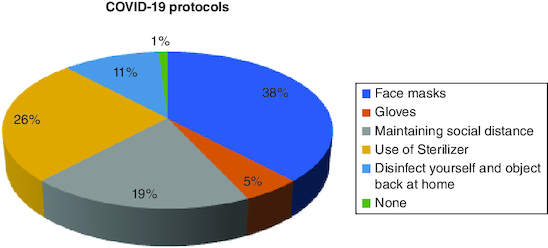
COVID-19 protocols during the lockdown.

During the COVID-19 lockdown, face masks were used by 38% (n = 83) of the participants, gloves by 5% (n = 11), 19% (n = 42) reported maintaining social distancing, 26% (n = 57) reported disinfecting themselves and objects when back at home and 11% (n = 25) were not following any COVID-19 protocols (Figure 1).

## Discussion

The COVID-19 pandemic has had a profound impact on lifestyles, leading to significant changes such as increased sedentary behaviors, decreased physical activity, disturbed sleep patterns and altered dietary habits [12,13]. We conducted our study during COVID-19 pandemic to evaluate these changes.

We identified a statistically significant distinction (p = 0.000) in dietary preferences, with individuals showing a tendency toward adopting a healthier diet. The consumption rate of a healthy diet increased notably from 25.6% to 48.1%. In a similar study conducted in Italy, the authors reported that throughout the COVID-19 lockdown, 37.4% of the study population reported an increase in the consumption of healthy foods (such as fruits, vegetables, nuts and legumes) while 29.8% of individuals reduced their intake of junk food [14]. In another study among young individuals conducted in Spain and Italy, a diminished adherence to healthy eating patterns, such as the Mediterranean diet, was identified [15,16].

We conducted an assessment of sleep duration changes among our participants and identified a highly significant difference (p = 0.000). The majority of participants (55.5%) reported sleeping 6–8 h before the lockdown, whereas, during the lockdown, this percentage decreased to 36.2%. On the other hand, the proportion of participants sleeping 8–10 h increased from 24.3 to 38.5%. Interestingly, the number of participants reporting less than 4 h of sleep dramatically decreased from 31.1 to 2.7% during the lockdown. Conversely, the percentage of individuals sleeping more than 10 hours increased from 3.6% to 11%. A study conducted by Carbonell *et al.* in the UK examined the impact of the pandemic on sleep patterns. The findings revealed that 69.4% of participants experienced alterations in their sleep schedule during the pandemic. Furthermore, 45.6% reported experiencing hypersomnia, 7.4% resorted to sleep medication, and 31.3% had sleep restrictions of less than 6 h per day [17,18]. Psychiatric disorders also associated with decreased sleep hours and use of anti-depressant drugs was major factors for reduction of sleep. This association between sleep reduction and use of drugs with psychiatric disorders was out of scope for this study but could be explored in further studies. The imposition of lockdown measures resulted in a substantial decline in physical activities among individuals.

The statistical analysis revealed a highly significant decrease (p = 0.000) in engagement with physical exercise. In a similar study conducted in Bangladesh, it was discovered that 37.9% of participants were physically inactive, while 38.3% had a moderate level of physical activity, and 23.9% exhibited high levels of physical activity. Notably, nearly 21% of individuals who were physically inactive had a significant amount of sedentary behavior, exceeding 8 h per day [19,20].

Our findings indicated that people decreased their workout frequency during the COVID-19 pandemic, as we discovered a statistically significant difference (p = 0.000) in workout habits before and after the pandemic. Huckins *et al.* also reported a decrease in a workout and an increase in a sedentary lifestyle during COVID-19 pandemic [21,22].

The COVID-19 lockdown has had an impact on smoking habits, as there was an observed increased trend of smoking among participants, with statistical significance (p = 0.0000). A study conducted in Turkey examined the influence of the COVID-19 pandemic on smoking addiction. The results revealed that 53.6% of participants maintained their smoking habits at the same level during the pandemic. However, among individuals with a low addiction level before the pandemic, there was an increase to 17.6 and 29.4% at moderate and high addiction levels, respectively, during the pandemic [20]. Another cross-sectional survey conducted in England demonstrated that the COVID-19 lockdown did not lead to a significant alteration in smoking prevalence, with rates remaining at 15.9% before the lockdown and 17.0% after. However, the lockdown was associated with notable increases in both quit attempts (29.1–39.6%) and successful cessation (4.1–8.8%) among individuals who had smoked in the past year [23].

Further analysis revealed no significant variance in the preference for specific food groups consumed (p = 0.845). We discovered that during the COVID-19 pandemic, individuals experienced an upsurge in the consumption of additional supplements. There was a statistically significant difference (p = 0.000), indicating a notable increase in the intake of warm water and drinks, as well as calcium, zinc and iron supplements. However, we did not observe any significant rise in the consumption of vitamins. A similar studies conducted in India has indicated that the fear of contracting COVID-19 during the lockdown prompted individuals to adopt certain healthy strategies, resulting in modifications to their daily routines. These strategies included engaging in meditation and consuming additional supplements [10,11].

Amidst the COVID-19 lockdown, it was observed that 38% (n = 83) of the participants utilized face masks, while gloves were used by 5% (n = 11). Additionally, 19% (n = 42) reported adhering to social distancing measures, 26% (n = 57) mentioned disinfecting themselves and objects upon returning home, and 11% (n = 25) did not comply with any COVID-19 protocols.

### Limitations of the study

This investigation was carried out in one public sector hospital and cross-sectional design which cannot determine the temporality of association. Also, because this study relied on self-reported data, it is susceptible to recall and social desirability biases. Sample size of study is small and suggested that further large sample size study need to be carried out for determine the incidence health risk.

## Conclusion

During the COVID-19 pandemic, participants living in Karachi experienced that the COVID-19 pandemic and associated restrictions and health orders likely impacted most heath domains and significant changes in their behavior and lifestyle. The findings emphasize the need for targeted interventions and public health initiatives to address these changes and promote healthier lifestyles during challenging times.
